# Gender dimensions of COVID-19 preventive policies: a global perspective

**DOI:** 10.11604/pamj.2022.41.199.29562

**Published:** 2022-03-11

**Authors:** Emery Manirambona, Annabel Killen, Don Eliseo Lucero-Prisno III

**Affiliations:** 1 College of Medicine and Health Sciences, University of Rwanda, Kigali, Rwanda,; 2 Medical Sciences Division, University of Oxford, Oxford, United Kingdom,; 3 Department of Global Health and Development, London School of Hygiene and Tropical Medicine, London, United Kingdom

**Keywords:** COVID-19, gender, male, female, preventive measures, health policy, health equity

## Abstract

The COVID-19 pandemic has had an impact on the global population not just from morbidity and mortality associated with SARS-CoV-2 infection, but also due to measures imposed upon populations to slow the transmission and prevent infections. Measures introduced by policymakers have included self-isolation of infective or potentially infective individuals, social distancing, travel bans, school closures, and mandatory face coverings. Most recently, the introduction of vaccination has been a key preventative measure encouraged by many governments. Considering gender differences in adherence to these measures is important to help guide future policymaking and targeting of advice. Differences also arise in how the preventative measures impact different genders. Some policies have caused greater harm to women, compounding existing problems such as inequality in the paid workforce, sexual- and gender-based violence, and inadequate maternal healthcare. Policymakers must consider the gender differences in response to preventive measures and creating effective and equitable policy.

## Commentary

**Introduction:** severe acute respiratory syndrome coronavirus 2 (SARS-CoV-2), the virus that causes coronavirus disease 2019 (COVID-19), poses a major threat to the global community. The first case was reported in China in December 2019, with the World Health Organization (WHO) declaring a pandemic on 11^th^ March 2020. Whilst the daily news of the pandemic has become less deafening, it has by no means ceased to threaten lives. As of 4^th^ October 2021, WHO had reported over 239 million cases worldwide, with a further 3 million new cases and 47 000 new deaths each week [1].

Policy measures were introduced in many countries as the infection first spread and were vital in reducing the respiratory virus spread between individuals. The compliance of individuals to drastic changes in their lifestyle necessitated by COVID-19 preventive measures has unsurprisingly been highly variable. Multiple factors such as educational level and co-habitation have been found to be linked to adherence. However, at present, no recent overview of the gender dimensions of the pandemic globally exists. Gender is a social and cultural construct, whereas sex is determined by biological aspects concerning anatomy, physiology and genetics.

Here, we focus on some ways gender appears to influence adherence to certain policies. We then switch our attention to how the impact of transmission-reducing strategies has unequal effects on each gender. Understanding these factors is critical in planning further interventions which are both practical and equitable. Due to limitations in current research, we discuss male-female gender differences only, although we acknowledge the need for policymakers to consider the full gender spectrum. This commentary makes generalised statements on gender dimensions based on available data; intersectional gender considerations, including ethnicity and economic position, are crucial in resolving inequalities and producing fair policies.

**Impact of gender on response to preventive measures:** given that gender encompasses social roles, gender differences in certain day-to-day behaviours undoubtedly exists. However, differing social roles do not fully explain the differences in adherence to protective measures reported. Many headlines have reported females being more likely to follow social distancing and hygiene protective policies. A study analysing two waves of a survey from March and April 2020 in eight high-income countries found women are more likely to perceive COVID-19 as a serious health problem. They also found that women are more likely to agree and comply with restraining public policy measures. These differences could not be accounted for by sociodemographic or employment characteristics [2]. Men also perform more poorly in analyses concerning hygiene measures. Studies have shown that males are less likely to follow advice concerning handwashing, use of disinfectant, cough hygiene, and avoiding touching their face. One study involving young people in Switzerland highlighted a non-migrant background and higher educational level as also being associated with non-compliance [3].

The pattern of face mask-wearing is conflicted in the literature. Whereas newspaper headlines have reported men are less likely to wear them, a study on participant-reported face mask-wearing found no significant difference in face mask-wearing between the genders [4]. They did, however, find significant differences in perception of face masks, with men more likely to avoid masks due to the feeling of infringement on their independence, whereas women more often cited discomfort as the reason. The potential stigma felt more greatly by men is backed up by a further American study, which found a higher proportion of males questioned (38%) than females (25%) would reportedly never wear a face mask. Differing results between studies is partly due to confounders such as location; the impact of this and time within the pandemic would be interesting.

Vaccination is another protective measure to which perceptions and uptake vary depending on factors including gender and location. Globally, women are less educated, limiting their access to accurate vaccine information and increasing vaccine hesitancy. In many areas of the world, females are not given autonomy over their health and may lack control over resources and mobility, preventing them accessing vaccination clinics. Caring and household responsibilities also act as a hindrance. Advice concerning vaccine safety has been very mixed for pregnant or breastfeeding women, and these groups have not been prioritized in vaccine research and development [5]. Despite the uncertainty surrounding vaccination in this group of women, the number of adverse effects from the COVID-19 vaccines appears to be similar between genders.

**Differential impact of preventive measures due to gender:** although most preventative measures such as stay-at-home rules have not distinguished between males and females, the impact of such public health policies on the genders has been unequal. Stay-at-home policies have increased the vulnerability of women to domestic violence. This is heightened by the loss of social and protective network contacts these restrictions cause, alongside increased relationship stressors due to loss of jobs and income. An increased SGBV (Sexual and Gender-Based Violence) rate became apparent early in the pandemic, with figures suggesting a 200% increase in crimes against women and children in Pakistan from February to March 2020 [6]. Increased tensions surrounding economic and health concerns are likely to increase the numbers of females at risk the longer the pandemic continues.

Access to healthcare during the pandemic has been a problem for both genders, yet perhaps the largest impact has been on maternal health, a vulnerable period in which medical attention is often time-critical. Examples have emerged from areas where compulsory quarantine and isolation have been enforced, such as the case of a woman in her first trimester suffering vaginal bleeding locked in a hotel room in Argentina. She was only able to access care after one of her children escaped through a window to get help. Whilst mental health issues have been on the rise across age groups and genders, again women who are pregnant or post-partum are significantly more vulnerable to depression than the general population. Coupled with isolation from family and support groups and the ongoing stressors, many pregnant women or new mothers are at increased risk of mental health issues arising.

Issues associated with poor access to maternal healthcare services during the COVID-19 pandemic are compounded by an increase in unplanned pregnancies. UNPFA (United Nations Population Funds) found 12,000,000 females in LMICs (Lower- and Middle-Income Countries) suffered disruptions to contraceptive services in the 12 months following the COVID-19 outbreak being declared a pandemic. This has resulted in at least 1,400,000 unexpected pregnancies [7]. The financial strain of an extra child is felt by all in the family, but the burden of health risks associated with pregnancy and childbirth, or unsafe abortion, is solely the mothers. Programs such as those aiming to reduce early marriage and teenage pregnancy have faced issues during the pandemic, with the closure of schools and increase in SGBV cases compounding the problem [8]. An increase in female genital mutilation globally has also been reported due to similar factors.

Gender-based gaps in domains such as economic participation, educational attainment and health had generally been narrowing steadily over the last 15 years. Nevertheless, in many places, the COVID-19 pandemic threatens such gains, with inequality in the work industry increasing in many countries. Countries including Canada, Japan, South Korea and the US saw increases in the gender pay gap from January to September 2020, alongside a reduced proportion of females in the workforce. In the USA, this has been attributed to the increase in family responsibilities of females during school closures, and the disproportionate impact of the pandemic on female-dominated work sectors such as hospitality and retail [9]. The reduced proportion, therefore, reflects both job losses and women exiting the workforce for unpaid domestic and care roles, work women already spent three times as much time on as men. A consideration globally is the risk of girls not returning to school post-closure, decreasing their future employment potential.

A discussion of the impact of virus control measures to control the pandemic must also consider the impact of the virus itself, as more cases arise when policies are inadequate. A systematic review of infection and mortality of healthcare workers worldwide found COVID-19 infection was more common in women (71.6%) [10]. Many factors likely contribute to this, such as the increased probability of female nurses involved in direct patient care, and the higher proportion of health and social workers worldwide being female (70%). The review found deaths were more common in men (70.8%), which is reflected in mortality trends across the general population. Despite this, morbidity after hospitalisation for COVID-19 appears most significant for women under 50; one UK study found this group were five times less likely to report feeling recovered at least three months post-discharge.

**Conclusions and recommendations:** this paper has reflected on some of the gender dimensions of COVID-19 preventive measures, as summarised in Table 1. Gender gaps which were already present-such as in research, employment and healthcare-have been widened by the pandemic. Solutions, therefore, are not quick fixes, but require ongoing investment into education and health infrastructure and political reform. Despite 70% of the global health workforce being female, only 25% of leadership positions in health are held by women. Balancing the gender divide in leadership across all domains could increase the relevance of policies towards meeting Sustainable Development Goal 5 (to achieve gender equality and empower all women and girls); the increase in SGBV, for example, suggests current laws and their enforcement surrounding this violence are failing women. Whilst many generalisations have been made in this commentary to allow the breadth of the topic to be discussed; it highlights that the pandemic has made a focus on gender equality even more crucial than ever.

**Table 1:** summary of gender dimensions addressed in the commentary alongside some recommendations to decrease the gender divide

		Proposed solutions
**Effect of gender on response to covid-19 preventative measures**	Better adherence by females to hygiene and distancing measures	Target resources towards reducing stigma in males and encouraging hygiene
	Differing perceptions to mask-wearing	Increase female education
		Increase autonomy of females
	More barriers to vaccine uptake among females	
**Ways preventative measures affected genders differently**	Increased domestic violence	Address gender data gap
		Prioritize pregnant women in vaccine research
	Greater impact of reduced access to healthcare on females	
		Considering intersectional gender dimensions in vaccination programmes
	Increased unplanned pregnancy causing health risks	
		Improve engagement of healthcare management and policy makers with women
	Greater drop in employment among females	Greater flexibility in employment and pay protection
	Increased mortality among males	
	Unclear morbidity impacts	
